# Determination of Endothelial Stalk versus Tip Cell Potential during Angiogenesis by H2.0-like Homeobox-1

**DOI:** 10.1016/j.cub.2012.07.037

**Published:** 2012-10-09

**Authors:** Shane P. Herbert, Julia Y.M. Cheung, Didier Y.R. Stainier

**Affiliations:** 1 Department of Biochemistry and Biophysics, Programs in Developmental and Stem Cell Biology, Genetics and Human Genetics, Cardiovascular Research Institute, University of California, San Francisco, San Francisco, CA 94158, USA; 2 Faculty of Life Sciences, University of Manchester, Michael Smith Building, Oxford Road, Manchester M13 9PT, UK

## Abstract

Tissue branching morphogenesis requires the hierarchical organization of sprouting cells into leading “tip” and trailing “stalk” cells [1, 2]. During new blood vessel branching (angiogenesis), endothelial tip cells (TCs) lead sprouting vessels, extend filopodia, and migrate in response to gradients of the secreted ligand, vascular endothelial growth factor (Vegf) [3]. In contrast, adjacent stalk cells (SCs) trail TCs, generate the trunk of new vessels, and critically maintain connectivity with parental vessels. Here, we establish that h2.0-like homeobox-1 (Hlx1) determines SC potential, which is critical for angiogenesis during zebrafish development. By combining a novel pharmacological strategy for the manipulation of angiogenic cell behavior in vivo with transcriptomic analyses of sprouting cells, we identify the uniquely sprouting-associated gene, *hlx1*. Expression of *hlx1* is almost entirely restricted to sprouting endothelial cells and is excluded from adjacent nonangiogenic cells. Furthermore, Hlx1 knockdown reveals its essential role in angiogenesis. Importantly, mosaic analyses uncover a cell-autonomous role for Hlx1 in the maintenance of SC identity in sprouting vessels. Hence, Hlx1-mediated maintenance of SC potential regulates angiogenesis, a finding that may have novel implications for sprouting morphogenesis of other tissues.

## Highlights

► Expression of *hlx1* is associated with angiogenic cell behavior in vivo ► *hlx1* selectively marks sprouting endothelial cells during zebrafish development ► Hlx1 is required for intersegmental vessel angiogenesis in zebrafish embryos ► Hlx1 cell-autonomously maintains endothelial stalk cell potential

## Results and Discussion

**Figure 1:**
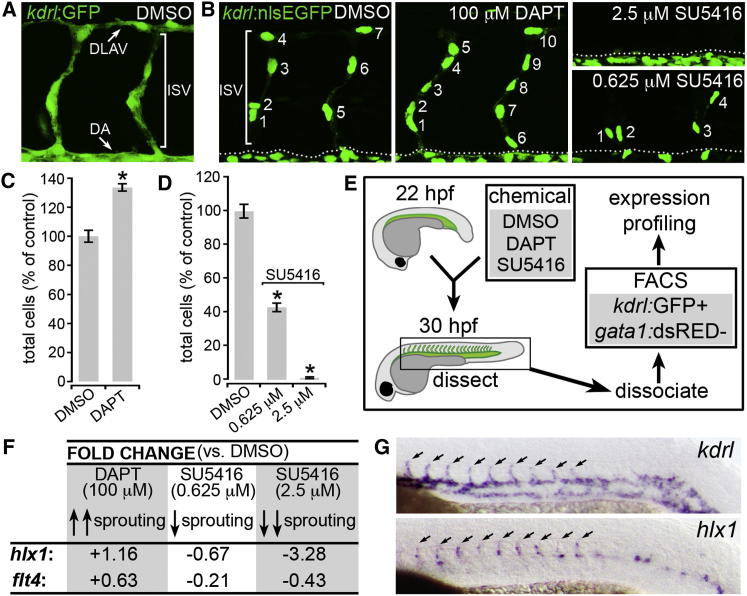
*hlx1* Expression Is Associated with Angiogenic Cell Behavior In Vivo (A and B) Lateral views of *Tg(kdrl:GFP)*^*s843*^ (A) or *Tg(kdrl:nlsEGFP)*^*zf109*^ (B) embryos at 30 hpf following incubation with either 0.4% DMSO, 100 μM DAPT, 2.5 μM SU5416, or 0.625 μM SU5416 from 22 hpf. Arrows in (A) indicate positions of the DA (dotted line in B denotes DA) and the forming DLAV, whereas white brackets in (A) and (B) indicate sprouting ISVs. (C and D) Quantification of ISV EC numbers at 30 hpf upon incubation of *Tg(kdrl:nlsEGFP)*^*zf109*^ embryos with either 100 μM DAPT (C) or the indicated concentration of SU5416 (D) (n = at least 21 embryos). A total of 100 μM DAPT augmented EC sprouting during ISV angiogenesis, whereas 2.5 and 0.625 μM SU5416 dose dependently disrupted EC-sprouting behavior. (E) Strategy for the identification of genes associated with EC-sprouting behavior in vivo. EC sprouting was pharmacologically manipulated prior to FACS-mediated isolation and transcriptome profiling of *kdrl*:GFP-positive ECs from dissected zebrafish trunks containing sprouting ISVs. Contaminating *kdrl*:GFP- and *gata1*:dsRed-double-positive erythrocytes were removed during FACS. (F) Fold change in EC *hlx1* expression upon incubation with the indicated chemical versus DMSO controls. EC *hlx1* expression was tightly correlated with the level of EC-sprouting behavior in vivo. (G) Whole-mount in situ hybridization analysis of the pan-endothelial marker *kdrl* or *hlx1* at 24 hpf. *hlx1* expression was enriched in sprouting ISVs (arrows). Error bars represent mean ± SEM. ^∗^p < 0.05 versus control, Student’s t test. See also Figure S1.

To identify previously unknown determinants of endothelial cell (EC) sprouting, we defined and exploited a pharmacological strategy for the manipulation of angiogenic cell behavior in vivo. Whereas high vascular endothelial growth factor receptor (Vegfr) signaling is known to promote tip cell (TC) specification, activation of the Notch receptor via its ligand Delta-like 4 (Dll4) represses the TC phenotype to promote stalk cell (SC) fate [4–6]. Conversely, suppression of Notch activity upon antagonistic interaction with its ligand Jagged1 promotes TC formation [7]. Hence, specification of TCs involves tight spatiotemporal control of Vegfr/Notch signaling [8]. Consequently, we hypothesized that the pharmacological manipulation of Vegfr/Notch signaling selectively during zebrafish intersegmental vessel (ISV) angiogenesis would enable the precise control of angiogenic EC behavior and sprouting-associated gene expression in vivo. In control dimethyl sulfoxide (DMSO)-treated *Tg(kdrl:GFP)*^*s843*^ embryos [9], green fluorescent protein (GFP)-expressing ECs sprout by angiogenesis at regular intervals from the first embryonic blood vessel, the dorsal aorta (DA), to form the ISVs. Nascent ISVs then connected with adjacent ISVs to form the dorsal longitudinal anastomotic vessel (DLAV) at 30 hr postfertilization (30 hpf; Figure 1A) [4, 10]. Quantification of EC numbers in sprouting ISVs using a nuclear-localized endothelial-specific enhanced green fluorescent protein (*EGFP*) transgene (*Tg(kdrl:nlsEGFP)*^*zf109*^ [11]) showed that ISVs at 30 hpf stereotypically contain three to four ECs, as previously reported [4, 11] (Figure 1B). However, using established pharmacological inhibitors of the Vegfr and Notch signaling pathways (SU5416 and DAPT, respectively), we were able to precisely manipulate sprouting EC numbers during ISV angiogenesis (Figures 1B–1D). EC sprouting was significantly enhanced upon incubation of embryos with DAPT from prior to ISV sprouting (22 hpf) to 30 hpf (Figures 1B and 1C), consistent with the EC hypersprouting phenotypes observed in the absence of Notch signaling [4–6]. In contrast, EC sprouting was entirely blocked in embryos incubated with high levels of Vegfr inhibitor (2.5 μM SU5416, Figures 1B and 1D), as previously observed [12]. Moreover, serial dilution of SU5416 (see Figures S1A and S1B available online) revealed that intermediate EC-sprouting phenotypes could be obtained upon partial inhibition of Vegfr (0.625 μM SU5416; Figures 1B and 1D). Hence, temporal disruption of Vegfr/Notch signaling during ISV sprouting allowed precise pharmacological control of angiogenic versus nonangiogenic EC behavior in vivo.

Exploiting these observations, we defined a novel strategy to identify genes functionally associated with EC sprouting (Figure 1E). *Tg(kdrl:GFP)*^*s843*^; *Tg(gata1:DsRed)*^*sd2*^ embryos were incubated from 22 to 30 hpf with compounds that either promoted (DAPT), fully repressed (2.5 μM SU5415), or partially repressed (0.63 μM SU5416) angiogenic cell behavior in vivo (Figures 1A–1D; Figure S1). Pharmacologically manipulated GFP-positive ECs were then isolated by fluorescence-activated cell sorting (FACS) and separated from GFP/dsRed-double-positive erythrocytes prior to comparison of their transcriptomes to DMSO-treated controls. Subsequent multifactorial comparison of expression profiles (see Experimental Procedures) identified 109 genes whose expression was tightly correlated with EC-sprouting levels, including *flt4*, the only known TC-enriched gene in zebrafish [4] (Figure 1F; Figures 1C and S1D). Surprisingly, the most SU5415/Vegfr responsive of these genes was a homeobox transcription factor gene, *h2.0-like homeobox-1* (*hlx1*) (Figure 1F; Figure S1C), which displayed an expression profile that was highly correlated with EC-sprouting levels (Figure 1F; Figure S1D). Furthermore, expression analyses revealed that compared to the pan-endothelial marker *kdrl* [13], *hlx1* was highly enriched in sprouting ECs in vivo (Figure 1G), suggesting a key role for Hlx1 during ISV angiogenesis. The mammalian ortholog of Hlx1 (HLX) was originally identified as a key determinant of mammalian liver, gut, and hematopoietic development [14–17]. Strikingly, *Hlx* null mice also display features of severe vascular dysfunction (edema, early lethality) [16, 17], and *HLX* was recently shown to influence expression of EC guidance cues in vitro [18]. However, the vascular function of HLX/Hlx1 in vivo is unknown.

**Figure 2:**
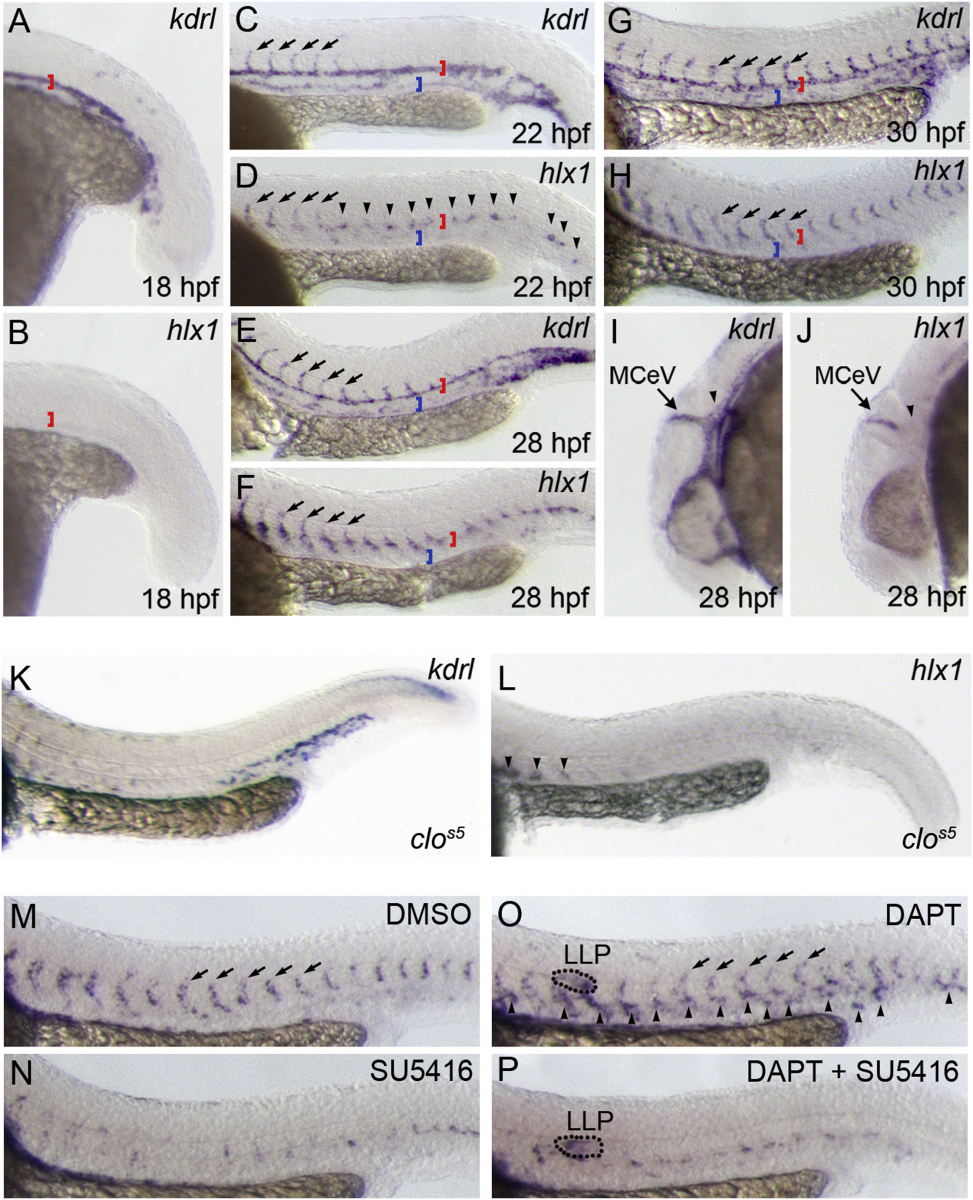
*hlx1* Expression Marks Sprouting ECs Whole-mount in situ hybridization analysis of *kdrl* (A, C, E, G, I, and K) or *hlx1* (B, D, F, H, J, and L–P) expression at 18 (A and B), 22 (C and D), 28 (E, F, I, and J), or 30 (G, H, and K–P) hpf in untreated WT embryos (A–J), *clo*^s5^ mutant embryos (K and L), or WT embryos incubated with DMSO (M), 0.625 μM SU5416 (N), 100 μM DAPT (O), or both 100 μM DAPT and 0.625 μM SU5416 (P) from 22 hpf (A–J; red brackets in A–H mark the DA, whereas blue brackets in C–H mark the cardinal vein). *hlx1* is not expressed during early vasculogenic assembly of the DA (B) but is initially expressed in the first-sprouting ISVs (arrows in C and D) and at regions of future angiogenic remodeling (arrowheads in D). At later stages *hlx1* expression is almost exclusively restricted to sprouting angiogenic ECs of the ISVs (arrows in E–H) and MCeVs (arrows in I and J) but is excluded from the adjacent nonangiogenic parental tissues of the DA (red brackets in A–H) and PHBC (arrowheads in I and J). (K and L) Both *kdrl* and *hlx1* expressions are reduced or lost in EC-deficient *clo*^s5^ mutants. Arrowheads in (L) indicate nonendothelial staining of the ventral somite. (M–P) SU5416-mediated suppression of EC sprouting abolished *hlx1* expression (N), whereas DAPT promoted ectopic *hlx1* expression in nonangiogenic tissues (arrowheads in O). DAPT-induced expression of *hlx1* was lost upon coincubation of embryos with the VEGFR inhibitor, SU5416 (P) (LLP, putative lateral line primordium).

To confirm an association of *hlx1* with angiogenic cell behavior in vivo, we assessed its spatiotemporal pattern of expression during zebrafish development (Figures 2A–2J). Compared with expression of the EC marker *kdrl* [13], *hlx1* expression was not detected in the first embryonic artery (DA), which forms by the process of vasculogenesis (red bracket in Figures 2A and 2B) [10, 12]. However, during ISV angiogenesis, *hlx1* expression was enriched in the first-sprouting ECs (arrows in Figures 2C and 2D). Expression of *hlx1* was also observed prior to ISV sprouting at discrete regions of future angiogenic remodeling within the DA (arrowheads in Figure 2D). At subsequent developmental stages *hlx1* became increasingly enriched in sprouting ISVs (Figures 2E and 2F) and was almost exclusively restricted to angiogenic ECs at 30 hpf (Figures 2G and 2H). Similarly, sprouting angiogenic ECs of the midcerebral veins (MCeVs) were also *hlx1* enriched (arrows in Figures 2I and 2J). However, *hlx1* expression was excluded from the adjacent nonangiogenic parental tissues of the DA and primordial hindbrain channel (PHBC) during ISV and MCeV angiogenesis (Figures 2G–2J). Importantly, vascular expression of *hlx1* was also absent in zebrafish *cloche* (*clo*^*s5*^) mutants that lack endothelial tissues [19], confirming expression of *hlx1* in sprouting ECs (Figures 2K and 2L). Furthermore, EC *hlx1* expression was reduced or lost upon the SU5416-mediated disruption of ISV sprouting (Figures 2M and 2N). In contrast, DAPT-induced EC hypersprouting promoted ectopic *hlx1* expression throughout the endothelium (arrowheads in Figure 2O), which could be blocked upon coincubation of embryos with SU5416 (Figure 2P). Finally, mature ISVs at stages after angiogenesis no longer expressed *hlx1* (data not shown). Hence, expression of *hlx1* exclusively marks sprouting ECs and represents a unique marker of angiogenic versus nonangiogenic ECs.

**Figure 3:**
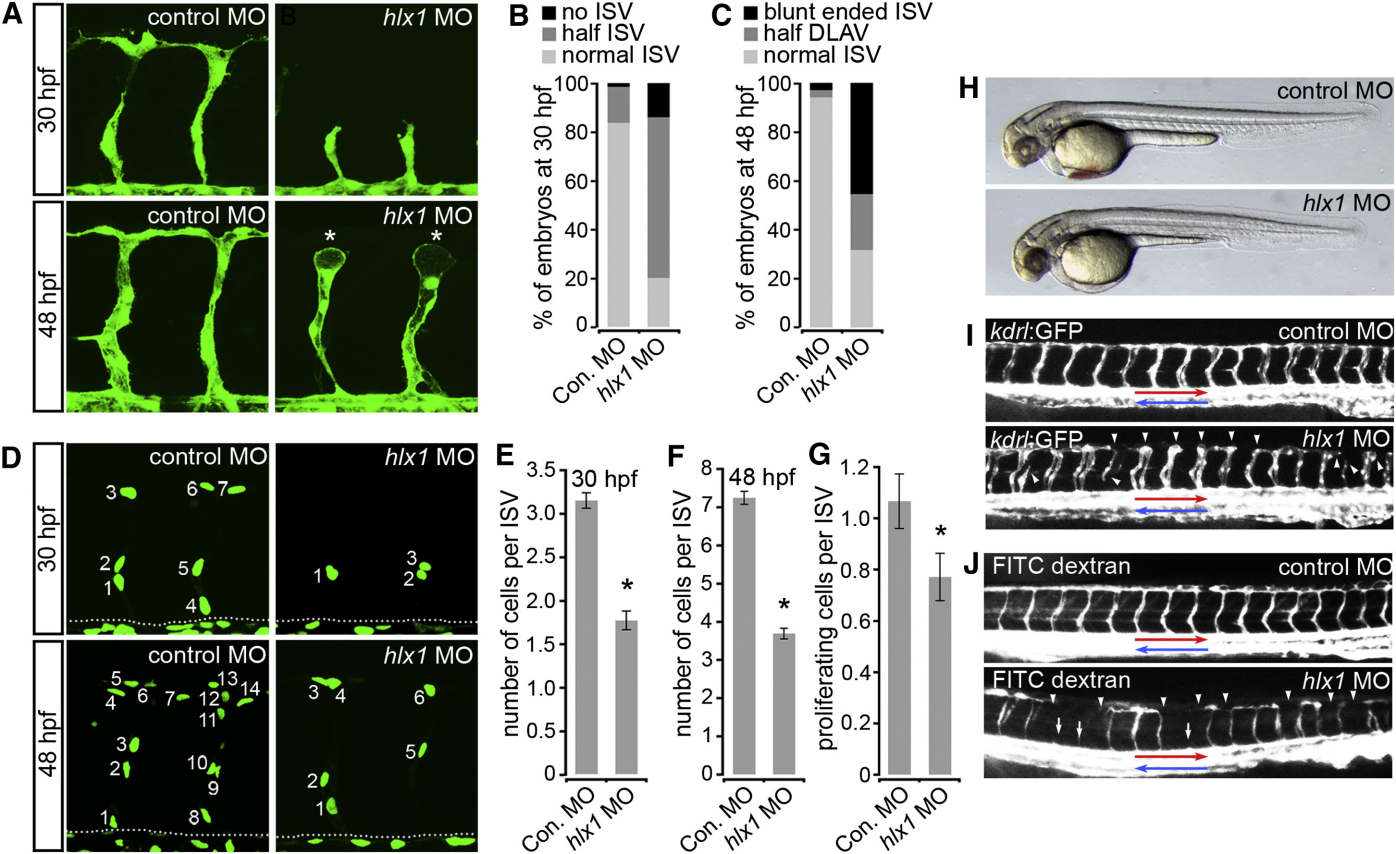
Hlx1 Is Required for ISV Angiogenesis In Vivo (A–F) Lateral views of *Tg(kdrl:GFP)*^*s843*^ (A) or *Tg(kdrl:nlsEGFP)*^*zf109*^ (D; dotted line represents position of the DA) embryos and quantification of ISV morphology (B and C; “half DLAV” refers to ISVs connected to only one adjacent ISV; n = at least 20 embryos) or ISV EC numbers (E and F; n = at least 15 embryos) at 30 (B and E) and 48 (C and F) hpf upon injection with either control MO (Con. MO) or *hlx1* MO. (G) Quantification of the number of dividing ECs per ISV following live imaging of control or *hlx1* MO-injected embryos from 19 hpf for approximately 13 hr (n = at least 30 ISVs from a total of 8 embryos). (H and I) Lateral views of control or *hlx1* MO-injected *Tg(kdrl:GFP)*^*s843*^ embryos at 48 hpf (red arrows indicate DA; blue arrows denote cardinal vein). Hlx1 knockdown disrupts ISV sprouting (as indicated by asterisks in A and arrowheads in I), limits EC incorporation into ISVs (D–F), and reduces EC proliferation (G). (J) Lateral views of control or *hlx1* morphants upon injection of FITC-dextran into the blood flow to assess vascular patterning at 48 hpf. White arrows indicate ISVs lacking blood perfusion. Red and blue arrows indicate arterial and venous blood flow, respectively. Arrowheads indicate ISVs that lack connections with adjacent ISVs. Blood flow through ISVs is disrupted upon Hlx1 knockdown. Error bars represent mean ± SEM. ^∗^p < 0.05 versus control, Student’s t test. See also Figures S2 and S3 and Movies S1 and S2.

To elucidate the function of Hlx1 during ISV angiogenesis, we injected *Tg(kdrl:GFP)*^*s843*^ and *Tg(kdrl:nlsEGFP)*^*zf109*^ embryos with either control morpholino oligonucleotides (MOs) or *hlx1*-targeting MOs that disrupted *hlx1* translation or exon-intron splicing (Figure S2). ISV sprouting in *hlx1* MO-injected (morphant) embryos was delayed at 30 hpf and severely disrupted at 48 hpf (Figures 3A–3G). In particular, ISVs in *hlx1* morphant embryos were predominantly stunted, failed to connect with adjacent vessels, and often remained blunt ended (asterisks in Figure 3A). Hlx1 knockdown did not notably affect embryo morphology (Figure 3H), arterial/venous differentiation of ECs (Figure S3A), assembly of the axial vessels (Figure 3I), or blood flow through the DA (red arrow in Figure 3J) and cardinal vein (blue arrow in Figure 3J). However, injection of embryos with *hlx1* MOs severely disrupted blood flow through the ISVs (Figure 3J), consistent with inadequate angiogenesis and reduced connections between adjacent vessels (Figures 3A and 3I). Importantly, perturbed ISV sprouting was associated with decreased incorporation of ECs into sprouting vessels (Figures 3D–3F) and reduced EC proliferation (Figure 3G). Hence, consistent with its specific expression in sprouting ECs, *hlx1* appears to be essential for ISV angiogenesis during zebrafish development.

**Figure 4:**
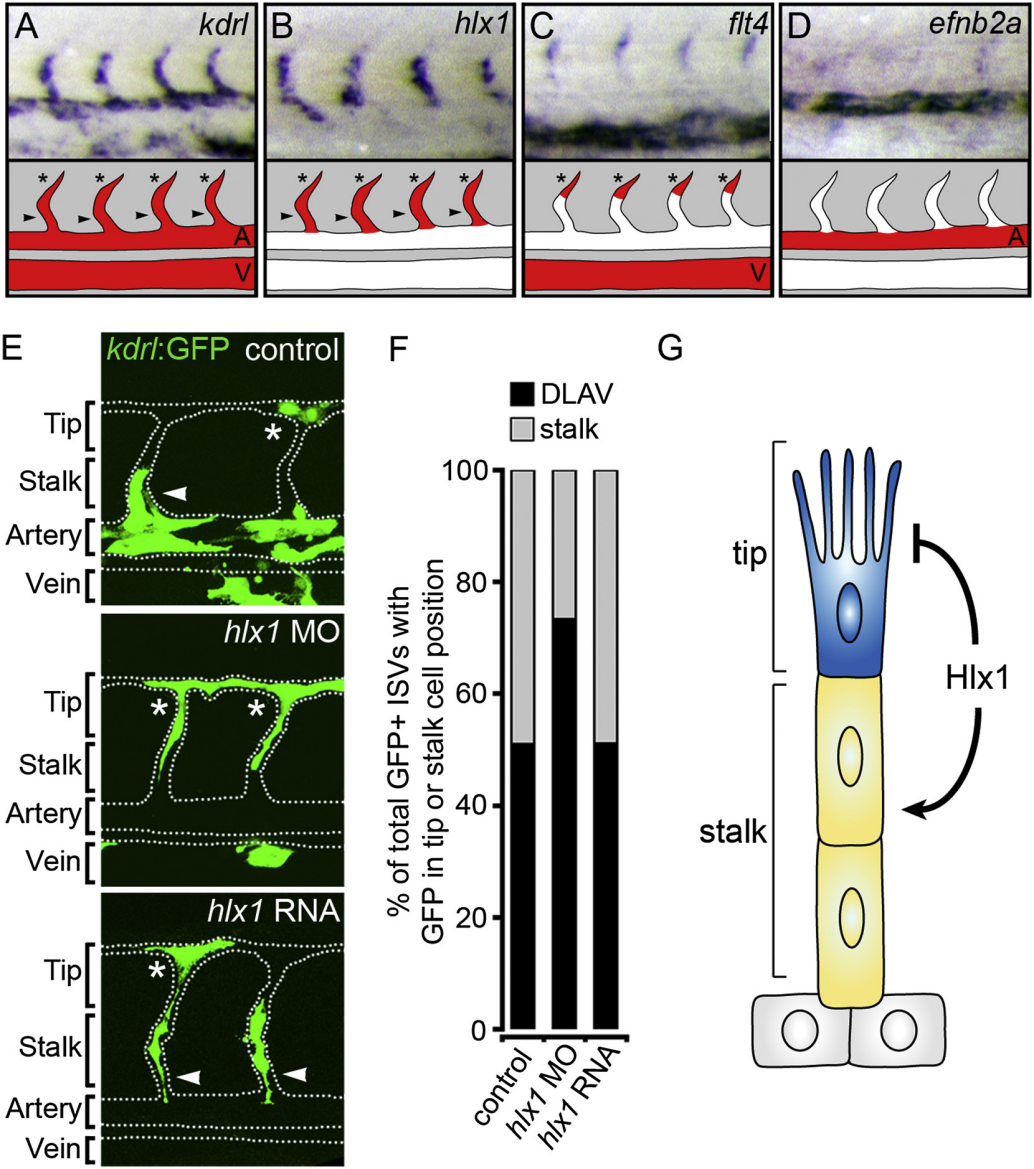
Hlx1 Cell-Autonomously Maintains Endothelial SC Potential (A–D) Whole-mount in situ hybridization analysis of *kdrl* (A), *hlx1* (B), *flt4* (C), and *efnb2a* (D) at 30 hpf and diagrams displaying the expression domain of each gene (V, cardinal vein; A, DA; arrowhead indicates SC; asterisks denote TC). Whereas *flt4* marks only TCs (C), *hlx1* uniquely marks SCs (and possibly TCs) of the sprouting ISVs (B) and is excluded from nonangiogenic ECs of the DA, which express high levels of *efnb2a* (D). (E) Lateral images of control MO, *hlx1* MO, or *hlx1* RNA-injected donor *Tg(kdrl:GFP)*^*s843*^ cells in nontransgenic hosts at 30 hpf (dotted line represents position of the WT vasculature). (F) Quantification of the incorporation of donor cells into the DLAV position (asterisks in E) or stalk position (arrowheads in E) of individual host ISVs (n = at least 44 ISVs; control = 19 embryos; *hlx1* MO, 17 embryos; *hlx1* RNA, 26 embryos). When *hlx1* is knocked down, ECs are lost from the ISV stalk position and accumulate in the DLAV position of sprouting ISVs. (G) Model summarizing the EC-autonomous function of Hlx1 during ISV angiogenesis. See also Figure S4, Movies S3 and S4, and Table S1.

Signaling via the Vegf-Notch axis promotes TC specification and behavior, at least in part, by inducing the TC-restricted expression of *flt4* [4, 13, 20]. Consequently, *flt4* morphant embryos display defects in EC sprouting similar to those observed upon Hlx1 knockdown [4]. However, TC-associated expression of *flt4* was comparable to controls in *hlx1* morphant embryos, indicating that TC specification was unaffected (Figure S3A). Furthermore, observations that EC sprouting in *hlx1* morphants was highly Flt4 dependent (Figures S3B and S3C) suggested that Hlx1-compromised ISVs still displayed TC behavior. Moreover, live-imaging analyses of *Tg(kdrl:nlsEGFP)*^*zf109*^ embryos revealed that the initial timing of TC migration was also unaffected in *hlx1* morphant embryos (Movies S1 and S2). However, the hierarchical organization of sprouting cells was disrupted in *hlx1* morphants versus controls (Movie S2). In particular, unlike in controls, sprouting ECs in *hlx1* morphants did not rapidly sort into leading TCs and trailing SCs but appeared to display prolonged competition for the TC position (see ISVs B and C in Movie S2). Consequently, we hypothesized that Hlx1 may alternatively influence SC identity. Consistent with this hypothesis, we observed that *hlx1* was uniquely expressed in the SC domain (as well as in TCs) during ISV sprouting (Figures 4A–4D). Whereas all vessels expressed the pan-endothelial marker *kdrl* (Figure 4A), *hlx1* expression was restricted to angiogenic-sprouting cells of the ISVs (Figure 4B). However, unlike expression of *flt4*, which was restricted to the TC domain of sprouting ISVs (Figure 4C), *hlx1* expression was expanded throughout the SC domain (Figure 4B). Moreover, *hlx1* was excluded from adjacent nonangiogenic ECs (or “phalanx” cells [21]) of the DA, which express high levels of *efnb2a* (Figure 4D) [22]. Other SC-enriched genes, such as *flt1* and *tie2*, are also expressed at high levels in adjacent nonangiogenic tissues [23, 24]. Hence, to the best of our knowledge, *hlx1* represents the first-identified discriminative marker of sprouting SCs versus nonangiogenic ECs.

SC-enriched expression suggested that *hlx1* influences SC identity. To elucidate the cell-autonomous role of Hlx1 in SC fate decisions at single-cell resolution, we transplanted cells from *Tg(kdrl:GFP)*^*s843*^ embryos into nontransgenic hosts. Previous studies have determined that the TC and SC potential of ECs can be assessed during zebrafish development based on the ability of transplanted ECs to contribute to the DLAV or ISV stalk position, respectively [4, 25, 26]. Hlx1 alone was not sufficient to induce SC fate because *hlx1*-RNA-injected donors contributed to the DLAV and ISV stalk positions with a similar frequency as controls (Figures 4E and 4F; Table S1). However, unlike controls, cells transplanted from *hlx1*-MO-injected donors frequently contributed exclusively to the DLAV position of ISVs (Tip, Figure 4E). Importantly, quantification of the positional fates of donor cells within ISVs confirmed that Hlx1-knockdown ECs were less likely to acquire SC fate, preferentially migrated to the DLAV position, and were found exclusively at the DLAV position of at least 42% of sprouting ISVs (Figure 4F; Table S1). Furthermore, live-imaging studies revealed that cells transplanted from *hlx1* morphant embryos, unlike controls, frequently exclusively occupied the leading TC position and did not contribute to ISV SCs (Movies S3 and S4). These data lead us to propose that Hlx1 functions cell-autonomously to reinforce and maintain SC potential during ISV sprouting (Figure 4G). Hence, Hlx1 regulates angiogenesis by influencing the outcome of EC competition for the TC position.

Previous work has primarily focused on defining the roles of VEGFR and Notch signaling in TC formation during new blood vessel sprouting [3–8]. Here, we show that Hlx1-mediated maintenance of SC potential appears to be critical for normal ISV angiogenesis in vivo (Figure 3). Whereas TCs express high levels of promigratory genes (such as *vegfr2* and *flt4*) [4, 6, 20] and are highly motile, SCs need to be characteristically less motile to maintain their position behind TCs. HLX was recently found to impede the migratory behavior of ECs in vitro by inducing the expression of repulsive guidance molecules such as *UNC5B* [18]. Hence, Hlx1-mediated repression of EC migration may be critical for determining SC positioning and functional blood vessel sprouting. In addition, our findings indicate that Hlx1 may also positively influence EC proliferation, because a decrease in cell divisions was observed in the ISVs of *hlx1* morphant embryos (Figure 3G). Moreover, this proliferation defect may account, at least in part, for the reduced number of ECs in the sprouting ISVs of *hlx1* morphants (Figures 3D–3F), which contrasts sharply with the hypersprouting phenotype observed in mosaic *hlx1*-deficient ECs (Figures 4E and 4F). Most importantly, our findings provide new evidence that reinforcement of SC identity and positional fate is critical for angiogenesis and implicate Hlx1 in this process. Hence, a fine balance between TC- and SC-inducing signals is crucial for the coordinated sprouting of new blood vessels. Considering the potential therapeutic implications of manipulating SC formation during pathological angiogenesis [27], future studies defining the downstream transcriptional network and precise cellular mechanisms of Hlx1 function in vivo will be of great importance. Furthermore, because the global mechanisms controlling angiogenesis and the branching morphogenesis of various epithelial tissues appear to be highly conserved [1], our findings raise the exciting possibility that analogous mechanisms may also control SC identity in other systems.

## Supplemental Information

Document S1. Figures S1–S3, Table S1, and Supplemental Experimental Procedures

Movie S1. Time-Lapse Imaging of *Tg(kdrl:nlsEGFP)*^*zf109*^ Embryos Injected with Control MO, Related to Figure 3 Embryos were imaged from 19 hpf for approximately 13 hr. ISV sprouting is initiated by a single endothelial tip cell that is either trailed by an endothelial stalk cell (see ISV B) or divides to form two cells that rapidly hierarchically organize into leading tip and trailing stalk cells (see ISVs A and C).

Movie S2. Time-Lapse Imaging of *Tg(kdrl:nlsEGFP)*^*zf109*^ Embryos Injected with *hlx1* MO, Related to Figure 3 Embryos were imaged from 19 hpf for approximately 13 hr. Multiple cells frequently sprout into newly forming ISVs and display defects in their hierarchically organization into leading tip and trailing stalk cells (see ISVs B and C).

Movie S3. Time-Lapse Imaging of Transplanted Cells from Control MO-Injected *Tg(kdrl:GFP)*^*s843*^ Donor Embryos, Related to Figure 4 Embryos were imaged from 20 hpf for approximately 16 hr. Donor cells are capable of contributing to both the tip and stalk cell position of sprouting ISVs.

Movie S4. Time-Lapse Imaging of Transplanted Cells from *hlx1* MO-Injected *Tg(kdrl:GFP)*^*s843*^ Donor Embryos, Related to Figure 4 Embryos were imaged from 20 hpf for approximately 16 hr. Donor cells frequently preferentially contribute to the tip cell position of sprouting ISVs.
