# Supplementary material for: Determination of Endothelial Stalk versus Tip Cell Potential during Angiogenesis by H2.0-like Homeobox-1
Source: Curr Biol. 2012 Oct 9;22(19):1789–94. doi: 10.1016/j.cub.2012.07.037 (PMC3471071; doi:10.1016/j.cub.2012.07.037)
Supplement: Document S1. Figures S1–S3, Table S1, and Supplemental Experimental Procedures [file mmc1.pdf]

Current Biology, Volume *22*

Supplemental Information

Determination of Endothelial Stalk versus Tip Cell Potential during Angiogenesis by H2.0-like Homeobox-1

Shane P. Herbert, Julia Y.M. Cheung, and Didier Y.R. Stainier

Supplemental Inventory

1. Supplemental Figures and Tables

Figure S1, related to Figure 1

Figure S2, related to Figure 3

Figure S3, related to Figures 3 and 4

Table S1, related to Figure 4

2. Supplemental Experimental Procedures

3. Supplemental References

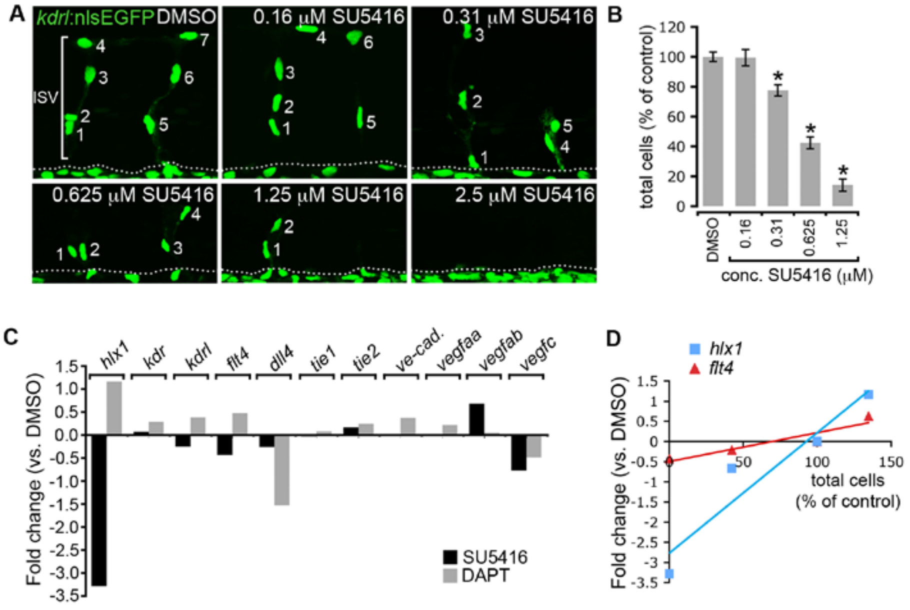

Figure S1. Pharmacological Manipulation of EC Sprouting and *hlx1* Expression, Related to Figure 1

(A-B) Lateral views of *Tg(kdrl:nlsEGFP)^{zf109}* embryos upon incubation with either DMSO or the indicated concentration of SU5416 from 22 to 30 hpf (A) and quantification of ISV EC numbers (B). SU5416 dose-dependently disrupted EC sprouting behaviour.

(C) Fold expression change of *hlx1* and known EC genes in expression profiles of ECs upon incubation with 2.5 µM SU5416 or 100 µM DAPT versus DMSO control. Expression of *hlx1* is highly SU5416 and DAPT responsive relative to other known EC genes.

(D) Correlation between the total number of sprouting ECs observed following chemical manipulation of ISV angiogenesis (taken from Figures 1C and D) versus the fold change in EC expression of *flt4* and *hlx1* (taken from Figure 1F). *flt4* and *hlx1* expression levels were highly correlated with EC sprouting. Error bars represent mean ± SEM. (**P* < 0.05 versus DMSO).

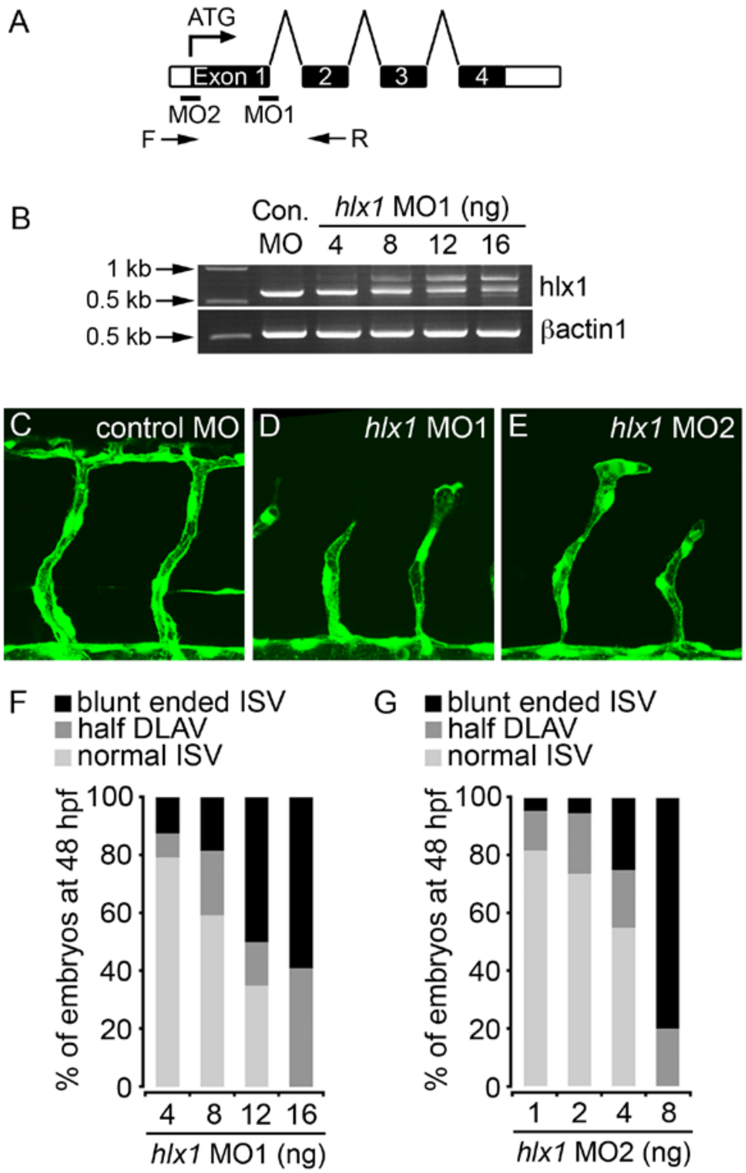

Figure S2. Knockdown of Hlx1 Using Gene-Targeted MO Reagents, Related to Figure 3

(A) Illustration of the *hlx1* intron-exon structure indicating location of complementary PCR primers (arrows; F = forward, R= reverse) and MO-targeted sites. MO1 targets the first exon-intron boundary whereas MO2 targets the ATG start site.

(B) RT-PCR of *hlx1* (using F and R PCR primers) or βactin1 using cDNA derived from embryos injected with either control MO or the indicated concentrations of MO1. MO1 dose-dependently alters *hlx1* intron 1 splicing, resulting in a PCR product size shift.

(C-G) Lateral views of *Tg(kdrl:GFP)*^{s843} embryos at 48 hpf (C-E) and quantification of the morphology of individual ISVs (F, G; half DLAV = an ISV connected to only one adjacent ISV; blunt ended ISV = an ISV with no connections to adjacent ISVs) upon injection of embryos with either control MO or the indicated concentration of MO1 (D; 12 ng) or MO2 (E; 4 ng). Hlx1 knockdown disrupts ISV sprouting. Throughout this study *hlx1* MOs were used as a combination of 4ng MO1 and 1ng MO2.

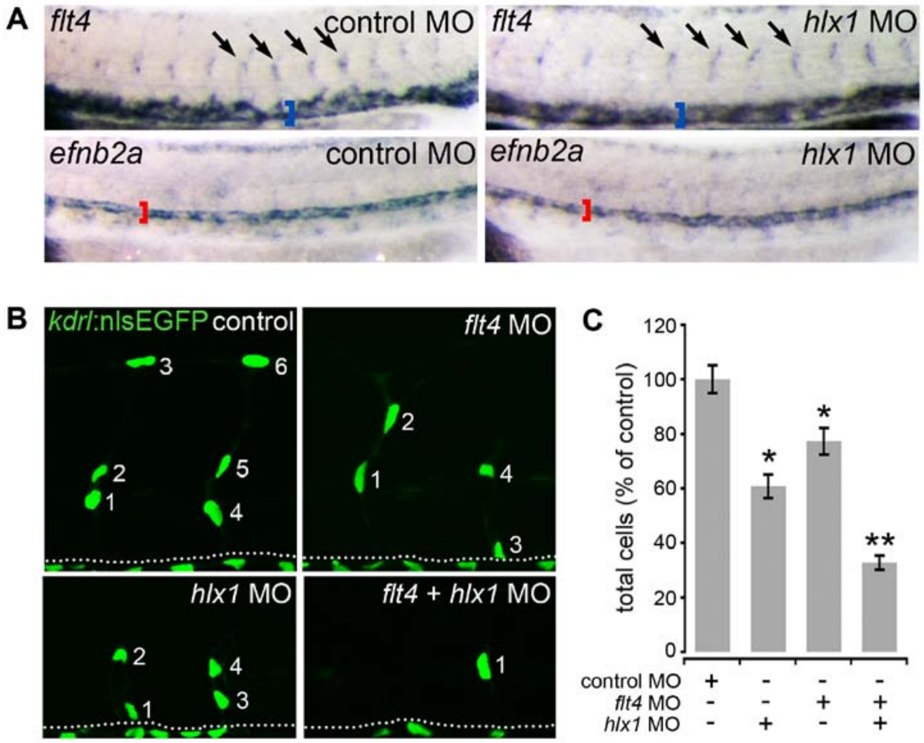

Figure S3. Flt4-Dependent TC Formation Appears Unaffected in the Absence of Hlx1, Related to Figures 3 and 4

(A) Whole-mount *in situ* hybridization analysis of *flt4* and *efnb2a* expression at 30 hpf in control and *hlx1* MO-injected embryos. Hlx1 knockdown did not detectibly disrupt *flt4* expression in sprouting TCs (arrows) and the CV (blue brackets) or *efnb2a* expression in the DA (red brackets).

(B-C) Lateral views of *Tg(kdrl:nlsEGFP)*^{zf109} (B; dotted line represents position of DA) embryos and quantification of ISV EC numbers (C) at 30 hpf upon injection of embryos with the indicated combinations of control MO, *flt4* MO and *hlx1* MOs. Upon Hlx1 knockdown EC sprouting remains highly Flt4-dependent. (n = at least 13 embryos). Error bars represent mean ± SEM. (*P < 0.05 versus control MO; **P < 0.05 versus *flt4* MO or *hlx1* MO).

Table S1. Positional Fates of Transplanted Donor ECs, Related to Figure 4

	Total ISVs with donor cells in SC position	Total ISVs with donor cells in TC position	% of donor cells in SC position of ISVs	% of donor cells in TC position of ISVs	Total embryos with GFP-positive ISVs	Total embryos receiving donor cells
**control MO**	**51**	**49**	**51**	**49**	**19**	**312**
***hlx1* MO**	**18**	**44**	**29**	**71**	**17**	**375**
***hlx1* RNA**	**65**	**68**	**49**	**51**	**26**	**312**

The total number of ISVs with transplanted cells in the SC or TC position and the overall % of donor cells residing in the SC or TC position expressed as a percentage of the total number of cells. In addition, the total number of host embryos with GFP-positive ISVs and the total number of host embryos originally receiving transplanted donor cells are listed.

Supplemental Experimental Procedures

Zebrafish Strains and Husbandry

Establishment and characterization of the *Tg(kdrl:GFP)*^{s843}, *Tg(gata1:DsRed)*^{sd2} and *Tg(kdrl:nlsEGFP)*^{zf109} lines have been described elsewhere [1-3]. Embryos and adults were maintained under standard laboratory conditions as described previously [4].

Pharmacological Treatments

Embryos were manually dechorionated and incubated with inhibitor from 22 to 30 hpf. The following chemical inhibitors were used in this study: SU5416 (0.625 µM; unless otherwise stated), DAPT (100 µM; Calbiochem) or combinations of the above.

Microscopy

For confocal microscopy, embryos were either fixed in 4% paraformaldehyde overnight at 4°C or incubated in 0.1% tricaine to anaesthetize (transplantation studies) prior to mounting in 1.5% low melting agarose in dishes. In the case of live embryos, agarose and covering media were also supplemented with 0.1% tricaine. Embryos were then imaged at 25°C with a 40x dipping objective on a Zeiss LSM5 Pascal confocal microscope. For time-lapse imaging studies, embryos were imaged at approximately 28°C with a 20x dipping objective on a Zeiss LSM 700 confocal microscope for 13-16h. For live-imaging movies, Zeiss VisArt*plus* software was used to generate clipping planes that removed contaminating signal from contralateral sprouting ISVs.

Isolation of Zebrafish ECs and Microarray Analyses

To isolate ECs from chemically manipulated 30 hpf embryos, zebrafish trunks and tails were dissected into ice cold Ca^{2+}/Mg^{2+}-free Hank's buffered salt solution (HBSS), washed four times in 1 ml ice cold Ca^{2+}/Mg^{2+}-free HBSS and dissociated in 2 ml TrypLE (Invitrogen) at 27.5° C for 30 min with regular agitation. Dissociation was inactivated by addition of 100 µl fetal bovine serum (FBS). Dissociated cell were subsequently isolated by centrifugation, re-suspended in 5 ml Ca^{2+}/Mg^{2+}-containing HBSS (with 5% FBS) and passed through 40 µm filters. ECs were collected upon re-centrifugation of dissociated cells, re-suspension in 0.5 ml Ca^{2+}/Mg^{2+}-containing HBSS (with 5% FBS) and FACS isolation of the *kdrl*:GFP-positive/*gata1*:DsRed-negative cell population.

FACS-isolated cells were sorted directly into lysis buffer and total RNA isolated using the RNAqueous-Micro kit (Ambion). Complementary DNAs were amplified, labelled with Cy3 (from DMSO-treated embryos) or Cy5 (chemical-treated embryos) and hybridized to the Agilent Zebrafish Gene Expression Microarray (V2) by Mogene Lc. The extracted data were normalized and quality controlled using GeneSpring GX software (Agilent). As RNA derived from angiogenic ECs represents a minimal proportion of the total EC RNA isolated we expected small gene expression fold-changes associated with differences in angiogenic behaviour *in vivo*. Hence, target genes were identified by performing a multi-factorial cross comparison between all experimental conditions to find genes whose expression correlated with angiogenic cell behaviour. Cutoffs for multi-factorial array analysis were set based upon the SU5416/DAPT-dependent fold-changes of *flt4*, a known TC-associated gene (2.5 µM SU5416 = -0.431x; 0.63 µM SU5416 = -0.206x; 100 µM DAPT = 0.63x).

Cloning of *hlx1* and Whole-Mount *In Situ* Hybridization

The zebrafish *hlx1 in-situ* hybridization construct was generated by PCR amplification of the *hlx1* ORF from cDNA and cloning of this fragment into pCR-Blunt II-TOPO (Invitrogen). For probe generation, pCR-Blunt II-TOPO *hlx1* was digested with BsaI and T7 was used for transcription. For whole-mount *in situ* hybridization, embryos were fixed in 4% paraformaldehyde overnight at 4°C and processed as described previously [5] using the following probes: *hlx1* (see above), *kdrl*, *flt4* and *efnb2a [4]*.

The zebrafish *hlx1* RNA expression construct was generated by subcloning of the pCR-Blunt II-TOPO *hlx1* ORF into pCS2+ via the BamHI and XhoI restriction sites. RNA was generated upon digestion of pCS2+ *hlx1* with SacII and SP6 was used for transcription. 300 pg of RNA was injected into embryos at the one-cell stage.

Morpholino Injections

Embryos were injected at the one-cell stage with 8 ng standard control-MO, 4-16 ng *hlx1* MO1, 1-8 ng *hlx1* MO2 or 4 ng *flt4* MO. All panels labelled *hlx1* MO refer to injection with a combination of 4 ng *hlx1* MO1 / 1 ng *hlx1* MO2. MO sequences were: 5'-GATTAAATTAGCGTCTTACCTCTCA-3' (*hlx1* MO1), 5'-AGCCGAACAATACGCAGTCCACAGG-3' (*hlx1* MO2), 5'-TTAGGAAAATGCGTTCTCACCTGAG-3' (*flt4*) [6]. All MOs were purchased from Gene Tools and were dissolved in water.

Cell Transplantation

Transplantations were performed as described previously [4]. Briefly, sense-strand-capped *hlx1* cRNA was synthesized with mMESSAGE (Ambion). Donor *Tg(kdrl:GFP)*^{s843} embryos were injected with 300 pg *hlx1* mRNA, 8 ng control MO or a combination of 1 ng *hlx1* MO2 and 4 ng *hlx1* MO1. After injections, donor and host embryos were manually dechorionated in agarose-coated dishes. Transplantations were performed at the mid-blastula stage and embryos maintained at 28°C until the indicated stage. Contribution of GFP-positive donor cells to the trunk vasculature was determined upon analysis and imaging of chimeric embryos with a Zeiss LSM5 Pascal confocal microscope. Contribution of cells to the TC or SC position of ISVs was calculated as a percentage of the total number of GFP-positive ISVs.

Supplemental References

1. Blum, Y., Belting, H.G., Ellertsdottir, E., Herwig, L., Luders, F., and Affolter, M. (2008). Complex cell rearrangements during intersegmental vessel sprouting and vessel fusion in the zebrafish embryo. Dev Biol *316*, 312-322.
2. Jin, S.W., Beis, D., Mitchell, T., Chen, J.N., and Stainier, D.Y. (2005). Cellular and molecular analyses of vascular tube and lumen formation in zebrafish. Development (Cambridge, England) *132*, 5199-5209.
3. Traver, D., Paw, B.H., Poss, K.D., Penberthy, W.T., Lin, S., and Zon, L.I. (2003). Transplantation and in vivo imaging of multilineage engraftment in zebrafish bloodless mutants. Nat Immunol *4*, 1238-1246.
4. Herbert, S.P., Huisken, J., Kim, T.N., Feldman, M.E., Houseman, B.T., Wang, R.A., Shokat, K.M., and Stainier, D.Y. (2009). Arterial-venous segregation by selective cell sprouting: an alternative mode of blood vessel formation. Science *326*, 294-298.
5. Thisse, C., and Thisse, B. (2008). High-resolution in situ hybridization to whole-mount zebrafish embryos. Nat Protoc *3*, 59-69.
6. Covassin, L.D., Villefranc, J.A., Kacergis, M.C., Weinstein, B.M., and Lawson, N.D. (2006). Distinct genetic interactions between multiple Vegf receptors are required for development of different blood vessel types in zebrafish. Proc Natl Acad Sci U S A *103*, 6554-6559.
