# Supplementary figures and images for: Determination of Endothelial Stalk versus Tip Cell Potential during Angiogenesis by H2.0-like Homeobox-1
Source: Curr Biol. 2012 Oct 9;22(19):1789–94. doi: 10.1016/j.cub.2012.07.037 (PMC3471071; doi:10.1016/j.cub.2012.07.037)

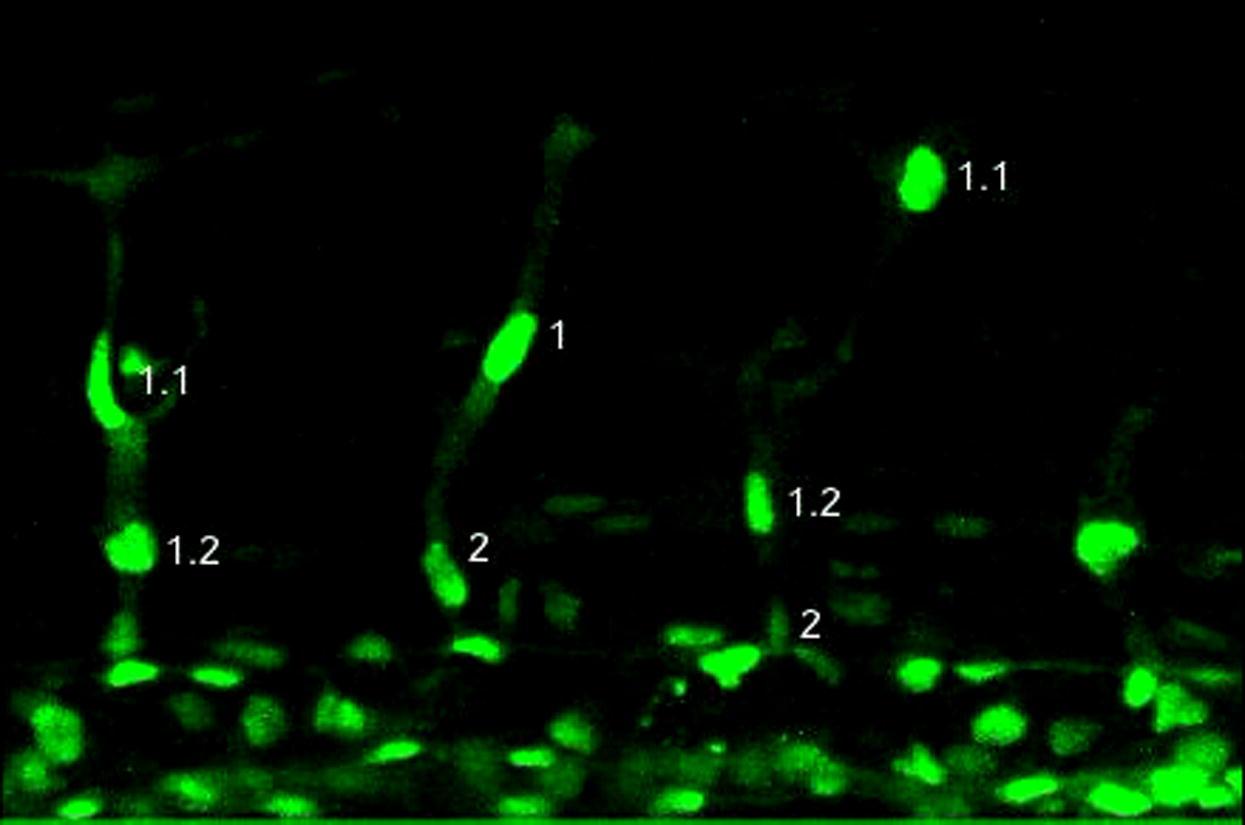

Supplement: Movie S1. Time-Lapse Imaging of Tg(kdrl:nlsEGFP)zf109 Embryos Injected with Control MO, Related to Figure 3 — Embryos were imaged from 19 hpf for approximately 13 hr. ISV sprouting is initiated by a single endothelial tip cell that is either trailed by an endothelial stalk cell (see ISV B) or divides to form two cells that rapidly hierarchically organize into leading tip and trailing stalk cells (see ISVs A and C). [file mmc2.jpg]

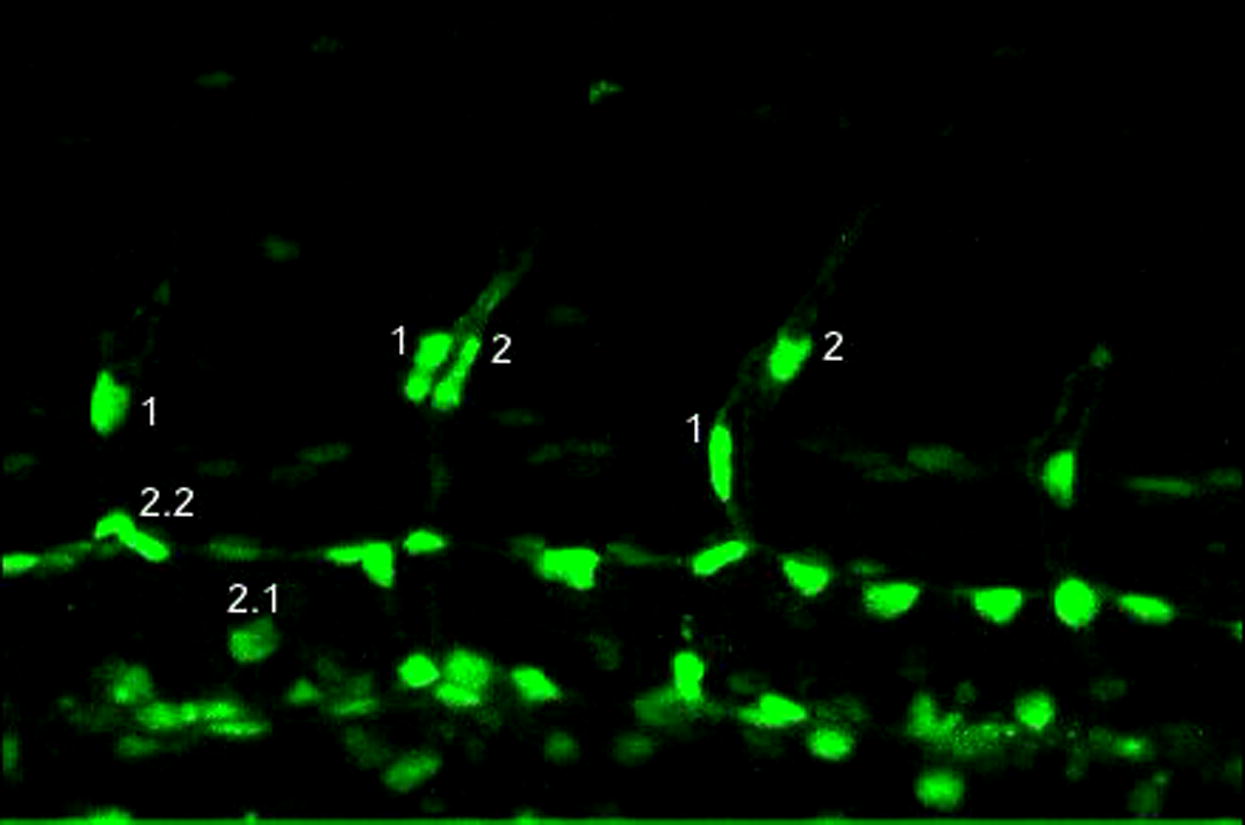

Supplement: Movie S2. Time-Lapse Imaging of Tg(kdrl:nlsEGFP)zf109 Embryos Injected with hlx1 MO, Related to Figure 3 — Embryos were imaged from 19 hpf for approximately 13 hr. Multiple cells frequently sprout into newly forming ISVs and display defects in their hierarchically organization into leading tip and trailing stalk cells (see ISVs B and C). [file mmc3.jpg]

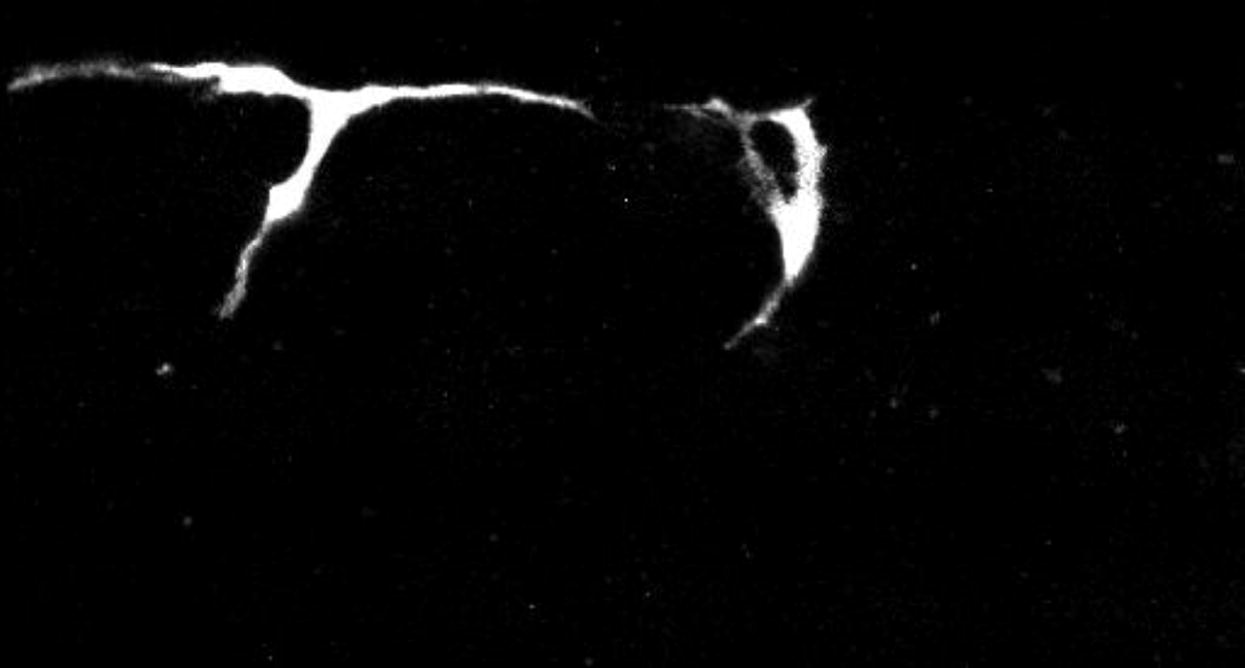

Supplement: Movie S4. Time-Lapse Imaging of Transplanted Cells from hlx1 MO-Injected Tg(kdrl:GFP)s843 Donor Embryos, Related to Figure 4 — Embryos were imaged from 20 hpf for approximately 16 hr. Donor cells frequently preferentially contribute to the tip cell position of sprouting ISVs. [file mmc5.jpg]
